# Prognostic significance of prenatal ultrasound in fetal arthrogryposis multiplex congenita

**DOI:** 10.1007/s00404-020-05828-4

**Published:** 2020-10-22

**Authors:** Brit Busack, Claus-Eric Ott, Wolfgang Henrich, Stefan Verlohren

**Affiliations:** 1 Department of Gynecology and Obstetrics, Universitätsklinikum Schleswig-Holstein, Lübeck, Germany; 2 Department of Genetics, Charité-Universitätsmedizin Berlin, Berlin, Germany; 3 Department of Obstetrics, Charité-Universitätsmedizin Berlin, Charitéplatz 1, 10117 Berlin, Germany

**Keywords:** Prenatal diagnosis, AMC, FADS, Fetal anomalies, Hydrops

## Abstract

**Purpose:**

Fetal arthrogryposis multiplex congenita (AMC) describes a heterogeneous disease entity characterized by multiple contractures affecting at least two different body areas. The aim of our study was to identify additional sonographic abnormalities in fetuses with AMC Type I–III associated with an unfavorable prognosis and to describe when those signs were first detected.

**Methods:**

This retrospective study included 41 pregnancies of suspected AMC diagnosed 1999–2017 at our tertiary referral center. The affected pregnancies were divided into the 3 AMC subgroups; the time of detection and outcome were analyzed. Prenatal sonograms, pediatric charts, genetic tests, and autopsy reports were studied.

**Results:**

Pregnancy outcome data were verifiable in 34 out of 41 cases; in 27 cases, AMC was confirmed. Hydrops was present in 50% of postnatally deceased fetuses, 53% of cases resulting in termination of pregnancy vs. 0% of the surviving 8 children. Absent stomach filling was found in 67% of the children with neonatal death. After subcategorization, the limb-involvement-only-group, 8% showed hydrops vs. 100% in system anomaly group vs. 70% in neuromuscular dysfunction cohort (*p* = 0.001). Scoliosis, nuchal edema, and absent stomach filling were significantly indicating for a neurological etiology.

**Conclusion:**

In addition to disease-defining sonographic findings, those with prognostic significance were identified. Hydrops, nuchal edema, scoliosis and absent stomach filling were associated with unfavorable outcomes implicating a neuromuscular etiology. This knowledge can help to predict the further course of the disease and support patient counseling.

## Introduction

Arthrogryposis multiplex congenita (AMC) is described by J.G. Hall as a symptom complex consisting of contractures of the joints of at least two different body areas [5, 8].

AMC (gr: arthron = joint, grypos = curved, multiplex = multiple, congenita = congenital, and -osis = disease without infection) occurs in 3000–5100 live births and is the descriptive term for a disease process that results in decreased fetal movement and subsequent joint stiffening [4, 13, 18]. The underlying etiology comprises 300–400 disease entities. The contractures are all present at birth, can be diagnosed by prenatal ultrasound, and usually do not aggravate during life if consequent physiotherapy is applied consistently [9]. Moessinger first defined the term “fetal akinesia deformation sequence” (FADS) in 1983 when he showed that curarized fetal rats show reproducible anomalies like IUGR, contractures, retrognathia, lung hypoplasia, short umbilical cord, and polyhydramnios. He emphasized that normal fetal movement is mandatory for normal intrauterine development [1, 6].

Regardless of whether the reason is a neuromuscular disease or a premature rupture of membranes in early pregnancy, the reduced fetal movement over 3 weeks leads to joint stiffness and muscular atrophy, and depending on the causative disease, there might be the craniofacial dysmorphia (retrognathia), lung hypoplasia, absent filling of stomach (due to lack of ability to swallow), scoliosis, nuchal edema, hydrops fetalis, pterygia, polyhydramnios, and intrauterine growth restriction (IUGR) [10, 16].

According to the Munich classification, modified by Hall, there are 3 types of AMC. Type I with mere joint affection as in amyoplasia, with a incidence of 1:10,000 the most common underlying cause of AMC, Type II with other system anomalies, and Type III with further neuromuscular involvement and lethal forms or intellectual disability like FADS [8, 16]. The detection rate of AMC by ultrasound is only 25% and the range of outcomes differs enormously due to the broad spectrum of underlying causes and complicates the counseling situation for future parents of fetuses with AMC [4].

The aim of our study was to identify additional sonographic abnormalities in fetuses with AMC Type I–III that are associated with an unfavorable prognosis and to describe when those signs were first detected.

## Methods

In this retrospective study, we included 41 pregnancies with sonographic suspicion of AMC, which referred to our tertiary referral center between January 1999 and January 2017, archived in viewpoint database. Inclusion criteria were joint fixations in at least two areas of the body which are obligatory for AMC and the suspicion of hypokinesia. Excluded were all isolated pes equinovarus or pathological fetal bone development.

Causes of referral were suspected anomalies for a second opinion or routine prenatal screening in the second or third trimester. The sonographic findings (images and clinical evaluation) were retrieved from Viewpoint (GE Healthcare GmbH. Solingen, Germany), and were reviewed together with personal files; for e.g., family history/birth mode, pediatric charts, physiotherapy reports, autopsy reports, prenatal genetic testing results from amniocentesis (AC) or chorionic villous sampling (CVS), MRI-scans and muscle biopsy, as well as laboratory results (including maternal myasthenia gravis antibodies). As a result of advancing tools for genetic analysis, increasing inheritance patterns have been identified over time; however, a specific genetic cause was identified in only three cases. A detailed fetal scan was performed and completed with pulse-wave-Doppler, 3D-ultrasound, and, if necessary, invasive procedures. In one case, amniotic drainage was required due to because of massive polyhydramnios.

Evaluation of hypokinesia was made by meticulous anamnesis followed by a detailed 45 min scan to observe carefully the fetal motor activity. All investigations were performed by experienced Fetal Medicine Specialists.

In 27 out of 41 cases, AMC was confirmed and detailed information on the outcome of pregnancy was available. The findings in prenatal ultrasound were first divided into AMC typical joint affections (extension/flexion) and additional organ/bone malformations such as nuchal edema, hydrops, scoliosis, lung hypoplasia, opisthotonus, abnormal profile (micro-/retrognathia), polyhydramnios, absent gastric filling, IUGR, and pterygia. To determine the time of detection, the cases were divided into 3 groups under 20 weeks of gestation (weeks), 20–24 weeks, and over 24 weeks. After grouping by gestational age, we analyzed and compared the sonographic findings with respect to different outcome [abortion, termination of pregnancy (TOP), live births, and neonatal death] to evaluate the prognostic relevance. According to the Munich classification, we compared the etiologies within the three groups to identify findings indicating system anomalies or neuromuscular dysfunction. Categorical variables were tested for significance using Chi-square test with SPSS Statistics version 25.0.

## Results

### Baseline characteristics

Mean presentation was at 22 + 6 weeks of gestation (min. 14 + 0 − max. 40 + 5 weeks). 14 pregnancies presented < 19 + 6 weeks, 6 between 20 + 0 and 23 + 6 weeks and 7 > 24 + 0 weeks. Three women were affected in two different pregnancies. There was one twin pregnancy with one affected child. Apart from that, all were singletons. Mothers were between 21 and 41 years of age. 13 fetuses were male, 6 fetuses were female, and the remaining 8 fetuses were not examined for their sex at the parents’ request but had a normal sex. We found one asymptomatic woman with maternal myasthenia gravis antibodies who had two affected fetuses in two consecutive pregnancies. Three women had autosomal dominant inherited forms of distal AMC. In 34 cases, we achieved detailed information concerning the outcome of pregnancy. In 27 cases, it was possible to assign the fetuses to AMC Type I–III; Type I with mere joint affection (Group A), Type II with other system anomalies (Group B), and Type III with further neuromuscular involvement and lethal forms or intellectual disability (Group C). 12 ended in TOP (mean 19 weeks of gestation), 2 times intrauterine fetal death resulted. 13 children were liveborn of which 5 died during neonatal period. Pediatric diagnosis showed 3 children with distal AMC (autosomal dominant) 4 AMC Type I Amyoplasia and one baby with Marden–Walker syndrome in the surviving group. In the 5 children who deceased after birth one had Kniest dysplasia, the other suffered from maternal Anti-AChR-Antibodies, Pterygium syndrome, one AMC Type I and one had Nemaline myopathy (confirmed by muscle biopsy). Severe AMC Type III in form of FADS was present in two subsequent pregnancies of a mother with asymptomatic Anti-AChR-antibodies. In the group of prenatally deceased fetuses, one fetus in 15 week aneuploidy with associated AMC was seen; furthermore, one fetus had FADS, one Nemaline myopathy, and one fetus with CMV associated AMC.

### Sonographic abnormalities in suspected AMC

**Fig. 1:**
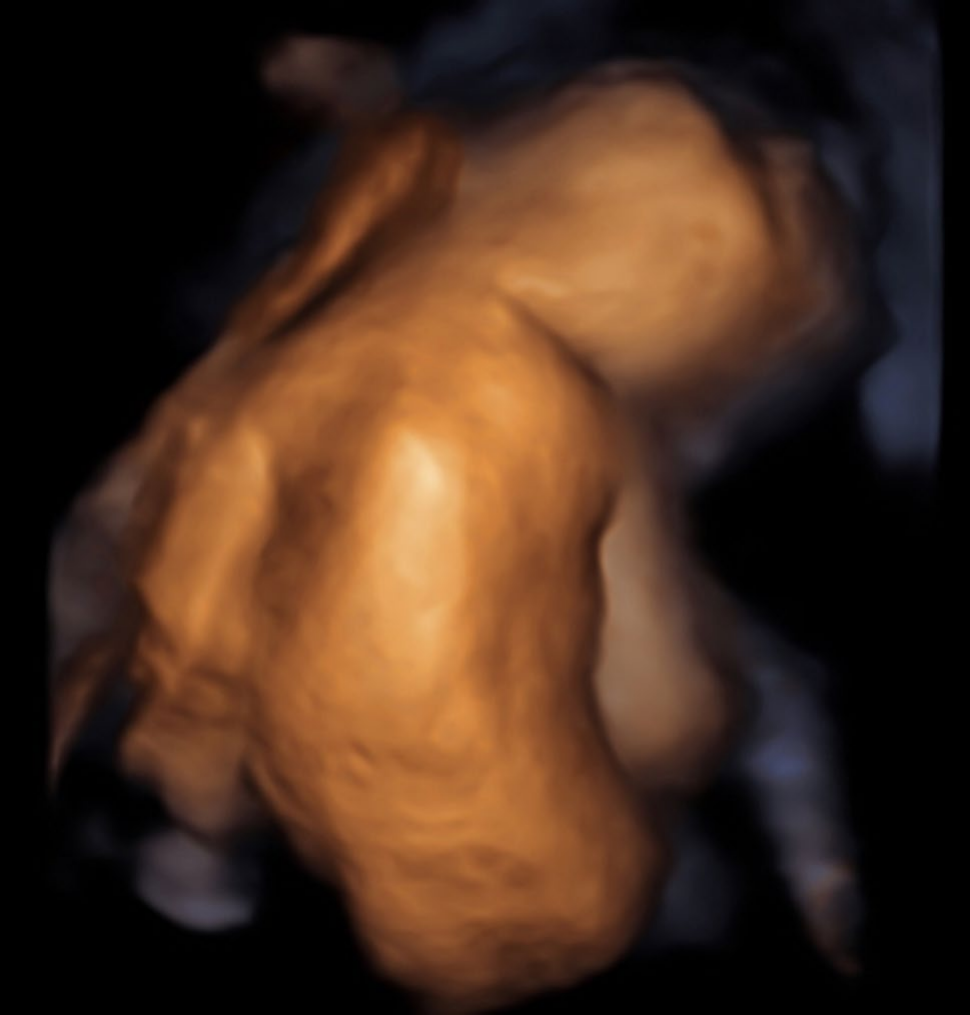
Scoliosis 26 weeks of gestation

**Fig. 2:**
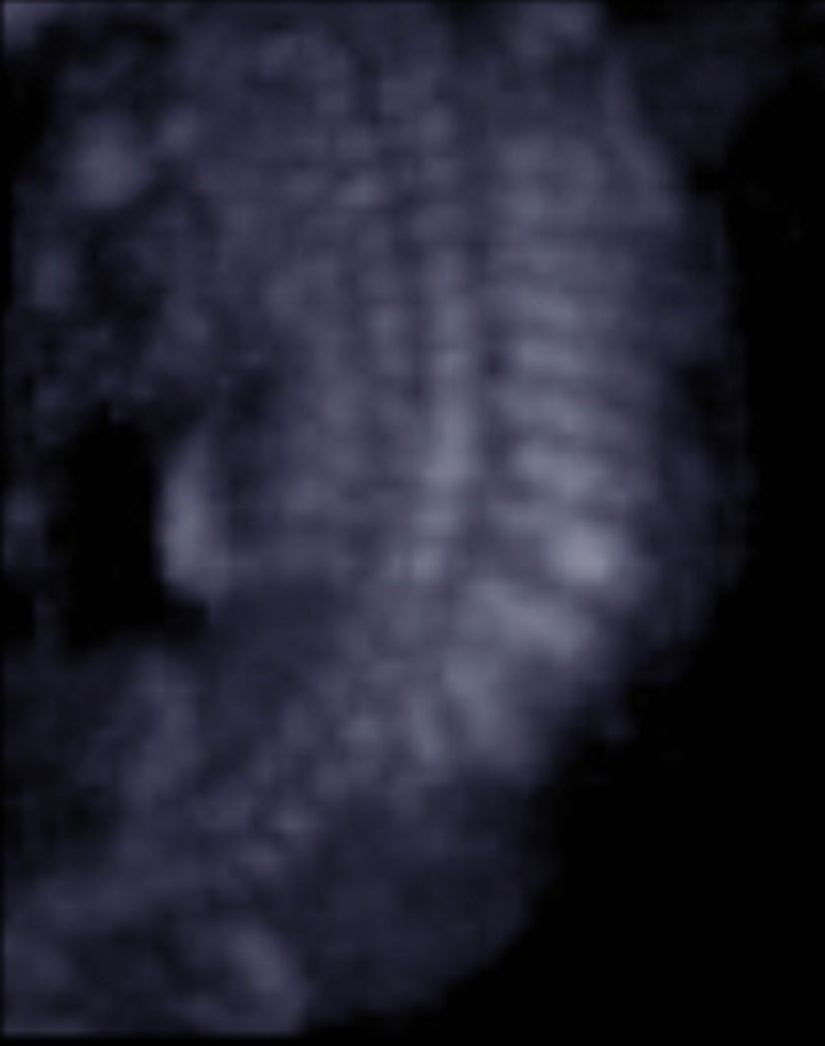
Scoliosis 18 weeks of gestation

**Fig. 3:**
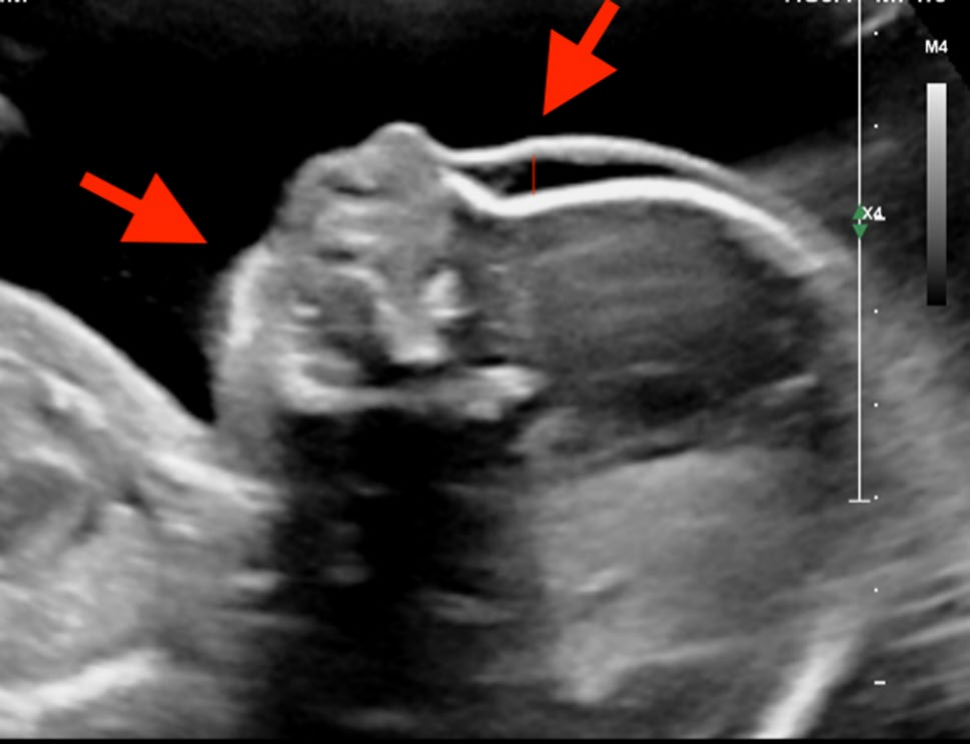
Prefrontal edema, retrognathia (arrows) 13 weeks

**Fig. 4:**
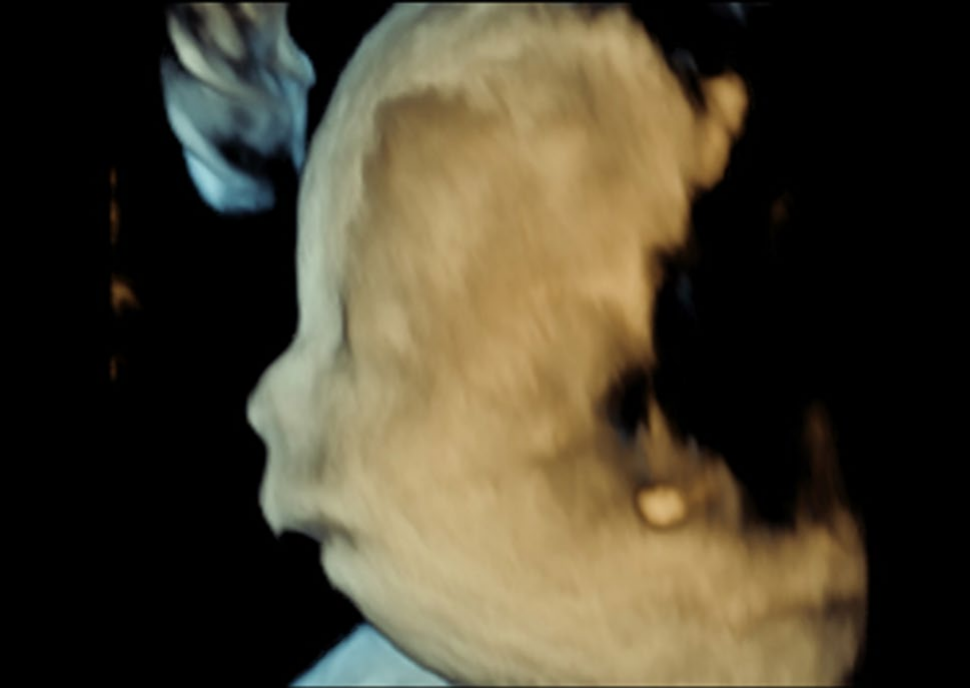
Prefrontal edema, retrognathia 13 weeks

All fetuses showed contractures, as per the definition of the disease. Of these, 85% showed malformations in all four limbs, 10% only in the upper extremities, and 5% only in the knee and ankle area. Elbow joint was fixed in 59%, 51% in flexion, and 10% in extension. Fixation of wrist concerned 41% of all cases, the majority 39% was fixed in flexion, and only 2% in extension. Abnormal finger fixations such as campodactyly or clinodactyly were visible in 37% of the fetuses. Affection of lower limbs was visualized in 70% in the knee joint, 48% in flexion, and 22% fixed in extension. Foot and ankle malformations were present in 63%. Deformations of the spine as scoliosis were seen in 15%, two of them as kyphoscoliosis, and in 2% as hyperextension of the spine in permanent opisthotonus (Figs. 1, 2). Profile abnormalities were observed in 26%. Two fetuses showed prefrontal edema, one flat profile and 4 had retro- or micrognathia (Figs. 3, 4). The majority had normal centile growth curves; 7% had reduced intrauterine growth. Polyhydramnios was present in 32% of pregnancies; in one case, amniotic puncture was necessary due to massive polyhydramnios. 33% had minimal or absent filling of stomach, which is related to a lack of swallowing ability. 15% showed hypoplastic thorax/lungs due to lack of diaphragmatic movement. Pterygia were seen in only one fetus and affected lower and upper limbs. Nuchal edema and hydrops were each present in 37% of the fetuses (Tables 1, 2).

**Table 1:** Prognostic relevance of sonographic signs (livebirth vs. antepartum death)

Sonographic findings	Livebirth *N* = 18	Mean 26 + 3 weeks	Died antenatally *N* = 16	Mean 18 + 0 weeks	*p* value
	Total	%	Total	%	
Pterygia	0	0.00	1	5.88	0.282
Hydrops	3	17.65	9	52.94	0.016
Nuchal edema	2	11.76	8	47.06	0.013
Thorax hypoplasia	2	11.76	2	11.76	0.900
Absent filling of stomach	5	29.41	5	29.41	0.824
Polyhydramnios	8	47.06	2	11.76	0.041
IUGR	2	11.76	1	5.88	0.618
Profile abnormalities	3	17.65	4	23.53	0.549
Scoliosis	0	0.00	4	23.53	0.024
Opisthotonus	0	0.00	1	5.88	0.282
Elbow fixed in flexion	10	58.82	8	47.06	0.746
Elbow fixed in extension	1	5.88	3	17.65	0.233
Wrist joint fixed in flexion	11	64.71	5	29.41	0.082
Wrist joint fixed in extension	1	5.88	0	0.00	0.339
Fingerfixation	8	47.06	3	17.65	0.110
Knee joint fixed in flexion	3	17.65	9	52.94	0.016
Knee joint fixed in extension	4	23.53	4	23.53	0.849
Pes equinovarus bilateral	8	47.06	6	35.29	0.681
Pes equinovarus unilateral	1	5.88	0	0.00	0.339
Rocker bottom	2	11.76	2	11.76	0.900
Ankle internal rotation	3	17.65	0	0.00	0.087

**Table 2:** Prognostic relevance (surviving children vs. ante- or postnatal death)

Sonographic findings	Survived neonatal period *N* = 12	Mean 24 + 3 weeks	Ante- or neonatal death *N* = 22	Mean 21 + 0 weeks	*p* value
	Total	%	Total	%	
Pterygia	0	0.00	1	4.55	0.453
Hydrops	0	0.00	12	54.55	0.001
Nuchal edema	0	0.00	10	45.45	0.005
Thorax hypoplasia	2	16.67	2	9.09	0.512
Absent filling of stomach	1	8.33	9	40.91	0.046
Polyhydramnios	3	25.00	7	31.82	0.677
IUGR	1	8.33	2	9.09	0.941
Profile abnormalities	2	16.67	5	22.73	0.676
Scoliosis	0	0.00	4	18.18	0.116
Opisthotonus	0	0.00	1	4.55	0.453
Elbow fixed in flexion	6	50.00	12	54.55	0.800
Elbow fixed in extension	0	0.00	4	18.18	0.116
Wrist joint fixed in flexion	7	58.33	9	40.91	0.331
Wrist joint fixed in extension	0	0.00	1	4.55	0.453
Fingerfixation	5	41.67	6	27.27	0.391
Knee joint fixed in flexion	2	16.67	10	45.45	0.093
Knee joint fixed in extension	2	16.67	6	27.27	0.486
Pes equinovarus bilateral	7	58.33	7	31.82	0.133
Pes equinovarus unilateral	1	8.33	0	0.00	0.169
Rocker bottom	2	16.67	2	9.09	0.512
Ankle internal rotation	1	8.33	2	9.09	0.941

### Gestational age-specific findings

Children presenting with eventful findings below 19 + 6 weeks exhibited hydrops in 63%, absent gastric filling in 38% and scoliosis in 25% of the cases. Polyhydramnios (25%) and thorax hypoplasia (13%) were less frequent in this group. Joint contractures predominantly affected the elbow joint in flexion (75%) and the knee joint in flexion (63%). Hydrops (*p* < 0.01), nuchal edema (*p* = 0.03), scoliosis (*p* < 0.01), and knee joint fixed in flexion (*p* = 0.001) reached statistical significance as compared to suspicious sonogram over 20 weeks of gestation.

The second group (20–24 weeks) had the highest rate of lung hypoplasia with 31% but no fetus presented with absent gastric filling, polyhydramnios, or IUGR. Contractures affected the wrist joint in flexion (54%) and bilateral pes equinovarus (54%).

**Fig. 5:**
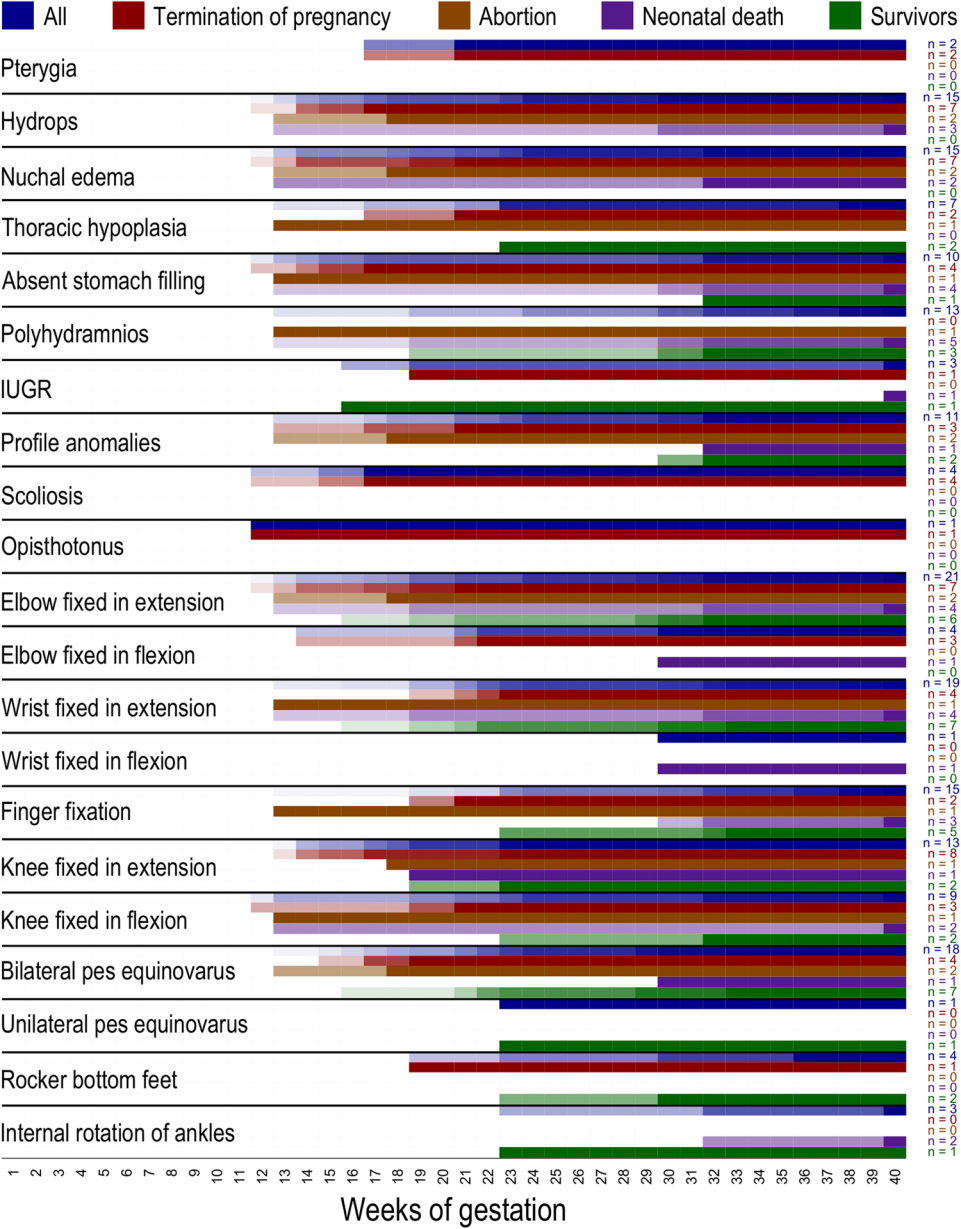
Overview of first recognition of ultrasound features. Features observed by ultrasound are listed on the left. Colors indicate groups of cases (all cases, terminations of pregnancy, miscarriages, neonatal deceased, and survivors)

When the first ultrasound abnormalities were detected after 24 + 0 weeks, 75% showed polyhydramnios and 33% absent stomach filling. Hydrops and nuchal edema were underrepresented with 25%. Finger fixations were observed in 67% of the fetuses. An overview of first recognition of ultrasound features is visualized in Fig. 5, the features observed by ultrasound are listed on the left. Colors indicate groups of cases (all cases, terminations of pregnancy, miscarriages, neonatal deceased, and survivors) (Fig. 5).

### Prognostic relevance of sonographic findings

We compared the group of liveborn children (*n* = 13) with those prenatally deceased (*n* = 14). Pterygia, scoliosis, and also opisthotonus were only present in group of IUFD and TOP. Hydrops, nuchal edema, polyhydramnios, scoliosis, and knee joint fixed in flexion were significantly prevalent in those fetuses dying during prenatal period (nuchal edema: *p* = 0.01, hydrops: *p* = 0.01, scoliosis: *p* = 0.02, and polyhydramnios: *p* = 0.04).

The group of all children who survived the neonatal period (*n* = 8) was compared with those who died before or after birth (*n* = 19). Findings that were present exclusively in the second group comprised pterygia, hydrops, nuchal edema but also absent gastric filling, scoliosis, and opisthotonus. There were significantly more fetuses with nuchal edema (*p* < 0.01), hydrops (*p* = 0.001), and absent gastric filling (*p* = 0.04) in the group that died ante- and postnatal. Contractures of elbow joint and knee were more common in the second group (elbow: 73% vs. 50%, knee: 73% vs. 33%) but without statistical significance.

Sonographic findings of surviving children (*n* = 8) were compared with those who died without any intervention pre- or postnatally (*n* = 7). Parameters like pterygia, opisthotonus, and scoliosis were only present in pregnancies which were ended by TOP. After exclusion of all terminations, the significance for an unfavorable outcome in nuchal edema (*p* < 0.01), hydrops (*p* < 0.01), absent gastric filling (*p* = 0.01), and also in polyhydramnios (*p* = 0.02) remained high. The surviving fetuses showed neither hydrops nor nuchal edema and polyhydramnios was with 25% relatively rare, only one fetus in this group had IUGR and absent gastric filling, and only two had profile abnormalities. Contractures predominated in wrist joint fixed in flexion (58%) and in ankle (58%).

After Munich classification group A (contractures limited to limbs), 8% showed hydrops vs. 100% in group B (system anomaly) vs. 70% in group C (neuromuscular dysfunction cohort) (*p* = 0.001). The absence of nuchal edema and hydrops was significant for group A (*p* < 0.001). Scoliosis was significantly indicative of a neurological etiology (*p* = 0.01), as well as absent stomach filling (*p* = 0.07) (Tables3, 4).

**Table 3:** Sonographic findings grouped after Munich classification (A: limbs-only; B: system anomalities; C: neuromuscular diseases)

Sonographic abnormalities (*N* = 41)	A *N* = 13	%	B *N* = 4	%	C N = 10	%	p B vs. A + C	p C vs. A + B
Pterygia	0	0	0	0	1	10		
Hydrops	1	8	4	100	7	70	0.001	0.001
Nuchal edema	2	15	3	75	5	50	0.05	0.05
Thorax hypoplasia	2	15	0	0	2	20		
Absent filling of stomac	2	15	1	25	6	60		0.07
Polyhydramnios	5	38	1	25	3	30		
IUGR	0	0	0	0	2	20		
Profile abnormalties	3	23	1	25	3	30		
Scoliosis	0	0	0	0	4	40		0.01
Opisthotonus	0	0	0	0	1	10		
Elbow joint fixed	7	54	3	75	6	60		
Wrist fixed	7	54	1	25	3	30		
Fingers fixed	5	38	1	25	4	40		
Knee joint fixed	8	62	3	75	8	80		
Ankle joint fixed	7	54	2	50	8	80

**Table 4:** Overview of sonographic findings, grouped after limb/neuromuscular involvement

	ID	First susp. US	Pter	Hydr	NuchE	ThHypopl	abs GF	Polyhydr	IUGR	Profile	Scol	Oph	Elbow	Elbow	Wrist	Wrist	Fingerfix	Knee	Knee	Ankle	Ankle	Ankle	Ankle		
													Flex	Ext	Flex	Ext		Flex	Ext	BPEV	UPEV	Rocker bot	Rot	Outcome I	Outcome II
AMC Type I	A4	13 5/7	–	–	+	–	–	–	–	+	–	–	+	–	–	–	–	+	–	–	–	–	–	TOP	AMC
	A5	14 1/7	–	+	+	–	+	–	–	–	–	–	–	+	–	–	–	+	–	–	–	–	–	TOP	AMC
	A1	19 4/7	–	–	–	–	–	+	–	–	–	–	+	–	+	–	–	+	–	–	–	–	–	Neonat. death	Distal AMC
	A7	19 4/7	–	–	–	–	–	+	–	–	–	–	+	–	+	–	–	+	–	–	–	–	–	Alive	Distal AMC
	A9	22 2/7	–	–	–	–	–	–	–	–	–	–	–	–	+	–	–	–	–	+	–	–	–	Alive	AMC Typ I
	A3	22 3/7	–	–	–	–	–	–	–	–	–	–	–	+	+	–	–	–	–	–	–	–	–	TOP	Distal AMC upper limbs
	A10	23 1/7	–	–	–	+	–	–	–	–	–	–	–	–	–	–	+	–	+	–	+	–	+	Alive	Distal AMC
	A11	23 1/7	–	–	–	+	–	–	–	–	–	–	–	–	–	–	+	+	–	+	–	–	–	Alive	Distal AMC
	A2	23 2/7	–	–	–	–	–	–	–	–	–	–	–	–	+	–	–	+	–	–	–	–	–	TOP	Distal AMC
	A12	30	–	–	–	–	–	+	–	+	–	–	+	–	+	–	–	–	–	–	–	+	–	Alive	AMC
	A8	32 1/7	–	–	–	–	+	+	–	+	–	–	+	–	–	–	+	–	+	–	–	–	–	Alive	Distal AMC
	A13	33 3/7	–	–	–	–	–	–	–	–	–	–	–	–	+	–	+	–	–	+	–	–	–	Alive	Distal AMC BPEV
	A6	36 1/7	–	–	–	–	–	+	–	–	–	–	–	–	–	–	+	–	–	–	–	+	–	TOP	AMC
AMC Type II	B4	13 3/7	–	+	+	–	+	+	–	–	–	–	+	–	+	–	–	–	+	–	–	–	–	Neonat. death	CMV
	B2	14	–	+	+	–	–	–	–	–	–	–	+	–	–	–	–	+	–	–	–	–	–	TOP	Chromosomopathy
	B3	18 4/7	–	+	+	–	–	–	–	+	–	–	+	–	–	–	–	+	–	+	–	–	–	Abortion	AMC
	B1	30 3/7	–	+	–	–	–	–	–	–	–	–	–	–	–	–	+	–	–	+	–	–	–	Neonat. death	Lysosomale disease
AMC Type III	C5	12 2/7	–	+	+	–	+	–	–	–	+	+	+	–	–	–	–	–	+	–	–	–	–	TOP	Maternal MG AB
	C6	13 1/7	–	+	+	+	+	+	–	+	–	–	+	–	+	–	+	–	+	+	–	–	–	Abortion	Maternal MG AB
	C10	14	–	+	+	–	–	–	–	–	–	–	+	–	–	–	–	+	–	–	–	–	–	TOP	AMC, Camptodakt, rocker bottom feet
	C9	15 3/7	–	+	–	–	+	–	–	–	+	–	–	–	–	–	–	+	–	+	–	–	–	TOP	AMC
	C3	17 5/7	+	+	–	+	–	–	–	+	+	–	+	–	–	–	–	+	–	+	–	–	–	TOP	Pterygia syndrome
	C7	17 6/7	–	+	–	–	+	–	–	–	+	–	–	–	–	–	–	+	–	+	–	–	–	TOP	Fowler syndrome
	C4	19 6/7	–	–	+	–	–	–	+	–	–	–	–	–	–	–	+	–	–	–	–	+	–	TOP	FADS
	C8	23 4/7	–	–	–	–	–	–	–	–	–	–	–	–	–	+	–	+	–	–	–	–	+	Alive	Marden–Walker (autos rec)
	C1	32 6/7	–	–	+	–	+	+	–	+	–	–	+	–	+	–	+	–	–	–	–	–	+	Neonat. death	Nemaline myopathy
	C2	40 5/7	–	+	–	–	+	+	+	–	–	–	+	–	+	–	+	–	+	–	–	–	+	Neonat. death	Nemaline myopathy

*Pter* pterygia, *Hydr* hydrops, *Nuch* nuchal edema, *ThHy* thoracic hypoplasia, *abs G* absent gastric filling, *Poly* polyhydramnios, *IUGR* intrauterine growth restriction, *Prof* profile, *Scol* scoliosis, *Oph* opisthotonus, *Elbo* elbow, *Wris* wrist, *Fing* fingerfixation, *Kne* knee, *Ankle:BPEV* bilateral pes equinovarus, *UPEV* unilateral pes equinovarus, *Rock* rocker bottom, *Rot* internal rotation

## Discussion

We were able to show specific sonographic findings helping to distinct between favorable and adverse outcomes in fetuses with suspected arthrogryposis multiplex congenita. The presence of nuchal edema, hydrops, scoliosis, and polyhydramnios were predictive parameters for intrauterine death versus live birth. Nuchal edema, hydrops, and absent gastric filling were predictive for antenatal or neonatal death versus survival of the neonatal period. Furthermore, we suggest that the presence of neck edema, hydrops, scoliosis, and a knee joint fixed in the flexion before the 20th week is very likely to lead to an unfavorable outcome. After subcategorization in the limb involvement only-group 7.6% showed hydrops versus 100% in system anomaly group and 70% in neuromuscular dysfunction cohort. Scoliosis was significantly indicating for a neurological etiology, but also absent stomach filling was prevalent in this group. Especially hydrops and nuchal edema but also scoliosis and absent stomach filling were associated with an unfavorable outcome and appoint to neuromuscular etiology. On the other hand, fetuses without these signs often had only limb involvement and good chances of survival.

In AMC, a multitude of underlying etiologies leads to the common denominator of multiple contractures [18]. The diverse sonographic findings in our population reflect the huge variety of diseases that can cause the AMC phenotype. This corresponds to the current literature. Paladini et al. stated that ultrasound diagnosis of FADS is feasible from 14 weeks of gestation onwards, whereas in our collective, the first suspicious ultrasound occurred in 12 weeks of gestation, caused by maternal anti-Myasthenia gravis antibodies [15]. A study by Hyett et al. showed 27 cases of fatal AMC, with 41% showing craniofacial anomalies such as high arched palate or micrognathia due to the lack of muscle pull at the attachment site that normally leads to bone remodeling [12]. In 2016, Hellmund et al. published a study on 79 fetuses with FADS of which 58% presented abnormal profiles [10]. In our population, profile malformations were relatively rare with 26% compared to the literature, maybe due to our mixed collective with mild and severe forms of AMC.

Noteworthy, the majority of fetuses had a normal growth along their percentile curve and intrauterine growth restriction (< 10th centile) was present in only 7% of pregnancies in fetuses with confirmed Nemaline myopathy and FADS. Fetuses affected before the 20th week exhibited significantly more nuchal edema, hydrops, scoliosis, and knee joint fixed in flexion, and let assume an association with a higher rate of terminations compared to fetuses over 20 weeks of gestation.

Hoellen et al. supposed that lung hypoplasia was obligatory for FADS and a good distinction parameter between AMC Type I with limb involvement only and AMC Type III [11, 16]. Thorax hypoplasia was visible in 13% < 19 + 6 weeks and in 20% > 20 + 0 weeks in mild forms of AMC as well as in the neuromuscular involvement group, suggesting that life-limiting lung hypoplasia has a low sonographic detection rate and mild forms can rarely also occur in fetuses with AMC Type I. It is recommended to combine ultrasound with information from magnetic resonance imaging (MRI) if lung hypoplasia is suspected [6, 11, 14]. Biophysical profile is not part of sonographic routine and is performed differently depending on the gestational age due to the fetal resting period [20]. In pregnancies up to 24 weeks of gestation, the systematic motor assessment can be performed during a 15 min ultrasound examination [3, 19]. If hypokinesia is suspected after 24 weeks, an ultrasound expert needs at least 45 min to examine the fetal movement of every limb area [8]. In our collective, observation of motor activity was made without systematic evaluation. For further prenatally distinction between the different AMC Types and suspected causes, it could be helpful to use a systematic motor assessment as performed by Donker et al. [3] or Tjon et al. [19].

Differential diagnosis of AMC includes ruling out trisomy 13, 18, and trisomy 8 mosaicism by AC or CVS [7]. In case of inconspicuous findings in karyotyping, a CGH microarray can be offered. Due to genetic heterogeneity, all modes of inheritance are possible. FADS (AMC Type III) follows an autosomal recessive inheritance, many distal AMC forms follow an autosomal dominant inheritance, some other forms are X-linked, and differential diagnoses even include mitochondrial disorders or sporadic entities such as amyoplasia [8, 9]. Multiple Pterygium Syndrome (Escobar syndrome), Potter Sequence, and Sheldon–Hall Syndrome are further differential diagnosis [8, 11]. Causes as PROM or multiple gestations but also positive family history are made by meticulous anamnesis (three-generation family history needed). Infections as CMV, coxsackie, or rubella can be diagnosed by maternal serum and by CVS; maternal antibodies against AChR should be excluded [17]. According to Hall, 50% of specific diagnoses are made after birth; in 75%, it is possible to evaluate the causing defect after age of 2 [8]. In a study by Hellmund et al. family history was positive in 20%. In our collective, family history was positive in 25% of the cases; no consanguinity was found. Two mothers with three affected children had autosomal dominant inherited distal AMC; in the other forms, autosomal recessive inheritance or sporadic occurrence was suggested. After exclusion of genetic disease, the remaining recurrence risk is 3–5%, in case of an unknown autosomal recessive inheritance 25% [8, 14]. One mother with two severely affected pregnancies was positive for maternal antibodies against the fetal gamma unit of ACh receptor as present in myasthenia gravis, but remained asymptomatic herself. In women with antibodies against the fetal ACh-R, the recurrence risk can be up to 100% [11]. Interdisciplinary counseling with geneticists, pediatricians, obstetricians, and orthopedists is essential [4]. Treatment sometimes is possible as in maternal acidosis or myasthenia gravis in context of studies [2, 17]. Due to the immobilization, the bone structure is altered and calcification is impaired. Consequently, the risk for trauma and perinatal bone fracture is high. Severely affected children should be delivered by elective C-section as the more gentle procedure and highly qualified neonatologists should be present to provide best pulmonary support [8]. Scoliosis and lung hypoplasia can make intubation and ventilation very difficult. In mild forms of AMC as in Type I, natural birth can be offered after good patient education.

In this study, we noticed a high number of terminated pregnancies. The decision to terminate is not necessarily associated with a fatal prognosis, but depends more on parental judgement after the have been confronted with evident malformations of their future child and several consultations with interdisciplinary recommendations. Terminations for medical reasons (for instance severe mental or/and physical disability of the fetus) are under certain conditions (§ 218 a Absatz 2 StGB) in Germany allowed until the onset of labor. It is essential to collect more data concerning the outcome to predict the further course of the disease and support parent counseling.

One limitation of our study is the overall low number of cases. In only 27 out of 41 cases, complete outcome data were available. Seven cases were lost to follow-up, and in 7 pregnancies, there was no confirmation or exclusion of AMC available. In case of prenatally suspected malformations, only follow-up can help to verify the diagnosis and to improve skills and counseling. As it is a retrospective study, the availability of methods for genetic testing has changed in the course of the recruitment period since 1999. Retesting the patients could be very purposeful, but even nowadays, there are many undetected genetic disorders, and in case of autosomal recessive inheritance, the recurrence risk can be up to 25%. Another weakness due to retrospective character of this study makes that not all sera of mothers were tested for myasthenia gravis. If fetal AMC is diagnosed, prenatally maternal antibodies should be excluded.

## Conclusion

We were able to define sonographic findings in fetuses with suspected AMC that were associated with an unfavorable outcome. Particularly nuchal edema and hydrops but also scoliosis, absent stomach filling, and polyhydramnios were associated with an adverse outcome. Those are all together more typical signs for AMC Type III (incl. FADS) and are rarely detected in AMC Type I. Although often cited, in our population, lung hypoplasia was not associated with an undesirable outcome. This knowledge plays a decisive role to provide adequate advice during a counseling situation of the future parents and to enable appropriate intervention early during pregnancy. In this context, it is essential to consider the pregnancy age regarding the specific ultrasound findings as they may occur at different gestational ages. Nevertheless, our study population also emphasizes that in fetuses with mild forms of AMC, the chances of survival are good. Since the contractures are non-progressive and the children usually are bright and resourceful, they are able to improve their mobility and live independently. Early recognition and adequate interdisciplinary treatment are indispensable to achieve the best initial conditions for affected individuals.
